# Male mate preference as an agent of fecundity selection in a polymorphic salamander

**DOI:** 10.1002/ece3.4298

**Published:** 2018-08-07

**Authors:** Kortney E. Jaworski, Matthew S. Lattanzio, Cari‐Ann M. Hickerson, Carl D. Anthony

**Affiliations:** ^1^ Christopher Newport University Newport News Virginia; ^2^ John Carroll University University Heights Ohio

**Keywords:** assortative mating, body size, clutch size, color polymorphism, mate choice, *Plethodon cinereus*

## Abstract

Color polymorphisms are associated with variation in other traits which may affect individual fitness, and these color‐trait associations are expected to contribute to nonrandom mating in polymorphic species. The red‐backed salamander (*Plethodon cinereus*) exhibits a polymorphism in dorsal pattern: striped and unstriped, and previous studies have suggested that they may mate nonrandomly. However, the mechanism(s) contributing to this behavior remain unclear. Here we consider the role that male preference may have in driving mating behavior in *P. cinereus*. We limit our focus to striped individuals because this morph is most likely to be choosy given their dominant, aggressive behavioral profiles relative to unstriped males. Specifically, we evaluated (a) whether striped males preferentially associate with females with respect to her dorsum color, size, and body condition and (b) if so, whether female traits are evaluated via visual or chemical cues. We also considered whether the frequency of another male social behavior, nose taps, was associated with mate preferences. We found that striped male *P. cinereus* nose tapped more often to preferred females. However, males only assessed potential mates via chemical cues, preferring larger females overall. Reproductive phenology data on a sample of gravid females drawn from the same population indicated that the color morphs do not differ in reproductive traits, but larger females have greater fecundity. Given our findings, we conclude that female *P. cinereus* are under fecundity selection, mediated by male preference. In this manner, male mating behavior contributes to observations of nonrandom mate associations in this population of *P. cinereus*.

## 1 INTRODUCTION

Many species exhibit discrete color morphs whereby differences in color coincide with variation in several fitness‐relevant physiological and behavioral traits (Brodie, 1992; Corl, Davis, Kuchta, Comendant, & Sinervo, 2010; Moreno, 1989; Reiter, Anthony, & Hickerson, 2014). As a result, color morphs are often inferred to reflect differences in mating tactics (Hurtado‐Gonzales & Uy, 2009), with their maintenance driven in part by sexual selection (Calsbeek & Cox, 2010; Lande, 1981). In particular, nonrandom mating has the potential to contribute to the evolution and maintenance of phenotypic diversity within a population and is likewise regarded as an important factor contributing to ecological divergence in color polymorphic systems (Gray & McKinnon, 2007). Numerous studies have shown that nonrandom mating is a widespread phenomenon across many taxa (reviewed by Jiang, Bolnick, & Kirkpatrick, 2013), including color polymorphic lineages (Pérez i de Lanuza, Font, & Carazo, 2013). Moreover, nonrandom mating has the potential to drive color assortment in the latter group (Pérez i de Lanuza et al., 2013; but see Sacchi et al., 2015). However, ever since Darwin (1859), most studies of mate preferences have been limited to the context of female choice. This apparent paradigm draws in part from the assumption that because females are gamete limited and may invest more into their offspring, males should engage in indiscriminate competition for female attention (Andersson, 1994). As a result, scant emphasis has been placed on the role of male choosiness in the mating process.

Evidence for male mate preference as well as its role in sexual selection has been growing over the past few decades (Amundsen & Forsgren, 2001; Bonduriansky, 2001; Marco, Chivers, Kiesecker, & Blaustein, 1998; Rosenqvist, 1990; Wearing‐Wilde, 1996). In these (and likely other) species, male preferences likely evolved to enable discrimination among females varying in reproductive quality (reviewed by Edward & Chapman, 2011). Male preference has also been documented in species exhibiting female preference (e.g., crayfish, Aquiloni & Gherardi, 2007; sticklebacks, Kraak & Bakker, 1998; Gouldian finches, Pryke & Griffith, 2007), revealing the potential for complex mating dynamics in color polymorphic species that would have been missed without consideration of male mate preference. Although male mate preference may be more common than once appreciated (see references above), the ability for males to exert sexual selection on female traits also requires that male choosiness coincides with differences in male quality (Fitzpatrick & Servedio, 2017). That is, theoretical models suggest that if choosy males are also socially dominant, they have the potential to impose sexual selection on females compared to other males in a population (Fitzpatrick & Servedio, 2017). In color polymorphic species, male color morphs often adhere to social dominance hierarchies whereby morphs differ in territorial and mating behavior (e.g., Sinervo & Lively, 1996; Thompson & Moore, 1991), such that only dominant, aggressive males can defend access to high‐quality (e.g., thermal resources, Calsbeek & Sinervo, 2002; prey types, Lattanzio & Miles, 2014) territorial resources. Compared to subordinate males, dominant males should therefore be of greater perceived quality to females and attract more potential female mates, providing them the opportunity to be choosy. Thus, if male mate preference occurs in color polymorphic species, only dominant territorial morphs are likely to both exhibit a nonrandom preference (due to higher female encounter rates) and contribute to selection on female phenotypes (due to the reproductive fitness benefits of access to high‐quality territorial resources).

**Figure 1:**
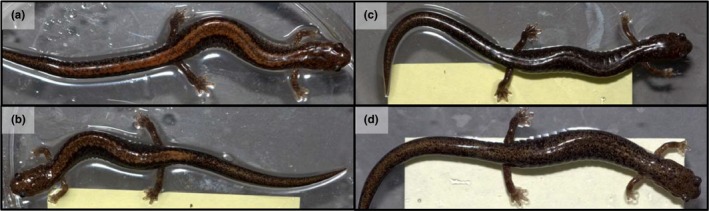
Representative images of the two morphs of the red‐backed salamander (*Plethodon cinereus*): (a–b) striped or (c–d) unstriped, highlighting some color variation within each morph. Both males and females exhibit these color morphs. Overall, despite some within‐morph variability in appearance, these two morphs significantly differ in color expression (based on RGB values; see Results, Figure 3) [Colour figure can be viewed at http://wileyonlinelibrary.com]

Red‐backed salamanders (*Plethodon cinereus*) represent an ideal system for investigating the role of male mate preference in the mating process. Two common color morphs occur in most populations throughout this fully terrestrial species’ range in northeastern North America (Petranka, 1998): striped and unstriped, referring to the presence or absence of a reddish mid‐dorsal stripe (Pfingsten & Walker, 1978; Figure 1). Previous studies support that these two morphs diverge along multiple ecological axes, including aspects of diet selection (Anthony, Venesky, & Hickerson, 2008; Stuczka, Hickerson, & Anthony, 2016), territorial behavior (Reiter et al., 2014), anti‐predator responses (Venesky & Anthony, 2007), and seasonal activity (Anthony et al., 2008; Moreno, 1989). These trait patterns support that morph coloration may also provide key information regarding individual quality during mate assessment (e.g., differences in prey or habitat use; Walls, Mathis, Jaeger, & Gergits, 1989). Recent field evidence also suggests that male and female salamanders spatially assort by color morph in the wild (Acord, Anthony, & Hickerson, 2013; Anthony et al., 2008).

Because there is limited evidence of female mate choice in this species (Acord et al., 2013), male mate preferences may be a primary mechanism behind this observed color assortment in wild *P. cinereus*. For example, although both sexes possess a vomeronasal sensory organ, only male organs hypertrophy during the breeding season (Dawley, 1992), implying a key role for this organ in males during courtship (Dantzer & Jaeger, 2007; Eddy, 2012; Marco et al., 1998). In addition, male *P. cinereus* also invest considerable energy toward reproduction via territory defense (reviewed in Mathis et al., 1995 and Petranka, 1998) and egg brooding (Cochran, 1911; Liebgold et al., 2006). However, choosiness should not be universal among males in this color polymorphic system. Specifically, the greater frequencies of territorial and dominant behaviors exhibited by striped males (e.g., Reiter et al., 2014) enable them to maintain access to higher quality or more abundant prey resources (Anthony, Jaworski, Messner, & Hickerson, 2017; Anthony et al., 2008; Paluh, Eddy, Ivanov, Hickerson, & Anthony, 2015) compared to unstriped males. These social and ecological attributes of striped males should attract a greater number of potential female mates compared to unstriped males (e.g., Acord et al., 2013). More importantly, dominant striped males do not simply associate randomly with their potential mates: for example, field‐based evidence supports that striped males are more likely to spatially associate with larger females (Acord et al., 2013). These considerations lay the foundation for nonrandom male mating preferences to evolve in striped males (e.g., Edward & Chapman, 2011), as well as their potential to impart selection on female traits (e.g., Fitzpatrick & Servedio, 2017).

In this study, we tested the hypothesis that dominant striped *P. cinereus* exhibit male mate preference with respect to multiple key female phenotypic attributes (color morph, body size, and mass). Our treatment of multiple female traits allows us to evaluate whether nonrandom male preference contributes to color assortment (female morph) as well as whether it also conforms to findings of previous studies inferring a preference for larger females (body size and mass, see Verrell, 1985, 1989). In addition, because mating dynamics often involve evaluation of cues deriving from multiple sensory modalities (Candolin, 2003), we ran separate experiments to evaluate male mate preferences for female visual and chemical cues, and explicitly tested whether male preferences overlapped among these distinct sensory modalities. Both visual and chemical cues are involved in the social interactions of *P. cinereus* and other terrestrial salamanders (visual cues, Anthony, Wicknick, & Jaeger, 1997; Eddy, Kiemnec‐Tyburczy, Uyeda, & Houck, 2012; Gergits & Jaeger, 1990; Jaeger, 1984; Jaeger & Forester, 1993; Kohn & Jaeger, 2009; chemical cues, Jaeger, Gollmann, Anthony, Gabor, & Kohn, 2016; Mathis et al., 1995). If striped male mating behavior is color assortative, then we expect males to prefer striped over unstriped females. Alternatively, males of other salamander species prefer larger females (e.g., Verrell, 1985), likely because larger body size or greater mass may signal greater fecundity (Tilley, 1968). To address this consideration, we assessed the body size‐fecundity relationship in our population of *P. cinereus* by rearing gravid females in the laboratory until egg deposition. Given these considerations, we predict that (a) larger females will lay more eggs and (b) males will exhibit an overall preference for larger females in our mating behavior experiments. Finally, because *P. cinereus* rely on both chemical and visual cues to communicate, we predict that male preference patterns will overlap in both experiments.

## 2 MATERIALS AND METHODS

### 2.1 Collection, housing, and morphology

Adult (>34 mm snout‐vent length [SVL], Sayler, 1966) male (*n* = 36, striped morph only) and visibly nongravid female (*n* = 98, 46 striped, 52 unstriped) red‐backed salamanders (*P. cinereus*) were collected by hand from a forested hillside in northern Summit County, OH (41.230282°N, −81.537762°W) during April–May 2013 (See Figure 1 for representative morph images). Sex was determined by visual inspection of snout morphology (Anthony et al., 2008). Individual salamanders were transported in separate containers to a laboratory in the Dolan Science Center at John Carroll University immediately following capture, where they were housed in separate glass Pyrex enclosures (11 cm diameter). We maintained a natural daylight cycle (~15‐hr light, 9‐hr dark) and a temperature range of 15–17°C in the laboratory for the duration of the study. Each container was filled with natural leaf litter substrate obtained from original capture location, and salamanders were provided food (natural leaf litter arthropods supplemented with wingless *Drosophila melanogaster*) regularly (every 10 days) until experiments began, approximately 5 months after collection. This acclimation period was necessary to allow all animals sufficient time to acclimate to our laboratory conditions and a constant diet prior to the fall breeding season. More importantly, this period allowed us to explicitly confirm female reproductive state for each female in our sample: overall, a total of 26 females (10 striped, 16 unstriped) were identified as gravid (confirmed visually by presence of enlarged mature eggs) during this acclimation period. These females were not included in our behavioral experiments and were instead retained for evaluation of their fecundity and reproductive phenology. We measured the SVL (mm, using calipers) and mass (g, using a digital scale) of each salamander on the day of capture, and re‐measured the mass of experimental animals (males and non‐gravid females) at the start of male mate preference experiments.

Male mate preference experiments were carried out from 30 September–2 December 2013, during the mating season of *P. cinereus* in Ohio (Anthony & Pfingsten, 2013). Two weeks prior to experimentation, all salamanders were switched to a laboratory diet of only fruit flies (*D*. *melanogaster*) and placed onto a dampened filter paper substrate. This diet switch served to control for the effects of variation in prey type and quality on female chemical cue composition, which is detectable by *P. cinereus* (Chouinard, 2012). Each female *P. cinereus* was provided 40‐50 *D. melanogaster* 6 days prior to initiating an experimental trial. We changed the filter paper substrate the day following feeding (i.e., 5 days before a trial) and removed any uneaten flies. This procedure allowed each female adequate time to mark her substrate with pheromones (Jaeger, 1981) prior to male preference trials and ensured that the substrate was not exposed to any direct prey cues that might also affect male preference behavior. We fed males every other day throughout the experiment. Each female was used in an experiment no more than once per week and individual males rested ≥36 hr between successive trials.

### 2.2 Color protocol

Evidence suggests that many amphibians have trichromatic color vision that enables detection of short‐, middle‐, and long‐wavelength light and therefore differentiation among red, green, and blue colors (Przyrembel, Keller, & Neumeyer, 1995). We therefore extracted quantitative color data in the form of red (R, long wavelength), green (G, medium wavelength), and blue (B, short wavelength) color channels from the dorsum of all females used in our behavioral experiments (*n* = 72 non‐gravid females). A single person (CDA) photographed each female at a fixed distance using a Nikon D5100 (16.2 megapixel) Digital‐SLR camera equipped with a Nikon SB‐400 Speedlight flash, under constant manual settings (flash: manual; camera: f/14, shutter 1/60, ISO‐100) and constant lab lighting conditions. All photographs were taken on the same day immediately following completion of both experiments. Although salamanders were held in captivity for several months prior to this point, we did not observe any color change and/or fading in our sample over this timeframe. Photographs were initially saved in Nikon Electronic Format (NEF) and later converted to linear Tagged Image File Format (TIFF) prior to analysis to retain high pixel quality (Stevens, Parraga, Cuthill, Partridge, & Troscianko, 2007). We used ImageJ (Abràmoff, Magalhães, & Ram, 2004) to extract the average RGB values occurring within three ~0.1 mm^2^ points along the mid‐dorsum region of each female: her shoulder, mid‐body, and base of tail. On striped individuals, we restricted our sampling to homogeneous red areas within the striped portion of the dorsum. We averaged loadings within each color channel across all three points to give a single R, G, and B value per animal (our repeatability of measurements across these points was 95.4%). We did not calibrate color data from our photographs because camera settings in our study were held constant (Davis & Grayson, 2008), unlike in other studies where camera processor attributes may vary between photos or comparisons are made across different data sets (Stevens et al., 2007). A Principal Component Analyses (PCA) was used to compress these RGB data into two orthogonal color variables (PC1 and PC2) for each female, following the methodology of others (Langkilde & Boronow, 2010; Mennill, Boag, & Ratcliffe, 2003; Vasquez & Pfennig, 2007). These variables therefore describe the two dominant axes of multivariate variation in dorsum color expression by female *P. cinereus*.

### 2.3 Visual experiment

**Figure 2:**
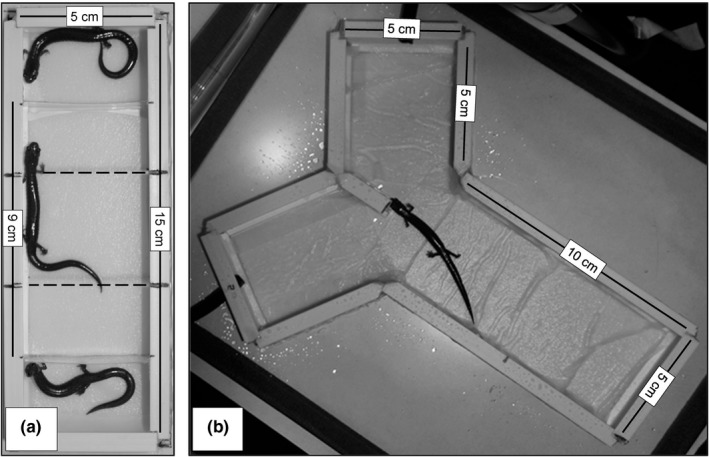
Birds‐eye perspective of the (a) Visual and (b) Chemical male mate preference arenas used to conduct our experiments. At the start of a trial, a male was initially placed in the center of the arena (Visual experiment) or at the far end of the long arm of the arena (Chemical experiment). Details on arena dimensions and experimental procedures are provided in Materials and Methods

We conducted an experiment to evaluate whether male *P. cinereus* can discriminate between females based on visual cues. In each preference trial, two females (one of each morph) were simultaneously presented to a male. Aside from this heteromorphic pairing constraint, females were selected at random for each trial. Each salamander was used in a maximum of four trials but was never paired with another individual (male or female) more than once. The preference arena for our visual experiment was a transparent rectangular enclosure (15 L × 5 W × 1.6 H, in cm) subdivided using clear styrene (0.03 cm thick) dividers into a central chamber for the focal male salamander (9 L × 5 W, in cm) and two female chambers (3 L × 5 W, in cm), one at each end of the arena (Figure 2a). Each chamber (central male and two female ends) was lined with spring‐water moistened filter paper to facilitate proper moisture levels. Prior to the start of each trial, the focal male was placed into the center of the male chamber of the experimental arena under an opaque cover for a 5‐min acclimation period.

Following this period, the cover was removed and one observer (KEJ) recorded behaviors (time in proximity [TIP, in s] and number of nose taps [NT]) of the male toward each female for 10 minutes using TrueBasic Event‐PC 3.0 data collection software (TrueBASIC, Inc.). Nose tapping behavior is an investigatory behavior by a salamander that conveys substrate‐borne odor molecules along its nasolabial groove to the vomeronasal organ (Brown, 1968). Time in proximity is calculated as the time (in seconds) in which the male is within the corresponding female's preference zone for each of the arenas (visual or chemical). Both behaviors are relevant for visual and chemical communication in *P. cinereus* (Chouinard, 2012; Gergits & Jaeger, 1990). Males could observe both females simultaneously, but clear dividers prevented physical interaction with the females. The arena was sealed with a glass lid and Vaseline^®^ to prevent air flow between chambers and thus also prevent transfer of female chemical signals to the male chamber during a trial (Kohn & Jaeger, 2009). We assigned a preference status to each female (preferred or not preferred) based on our estimate of the total TIP to each female (within nearest 3 cm of a female chamber). Time spent in proximity has been shown to be a useful indicator of mating preferences in a diverse array of taxa, including fish (Amundsen & Forsgren, 2001; Kodric‐Brown, 1989), amphibians (Liao & Lu, 2009; Verrell, 1985), birds (Amundsen, Forsgren, & Hansen, 1997), and mammals (Charpentier, Crawford, Boulet, & Drea, 2010; Szykman et al., 2001). We therefore considered the female having the greatest TIP to be the preferred female in that trial. All trials were conducted under constant, low light conditions (40‐W incandescent light reflected off a white wall; Kohn & Jaeger, 2009). At the end of each trial, we thoroughly cleaned the experimental arena with 70% ethanol prior to re‐use.

### 2.4 Chemical experiment

We also conducted an experiment to evaluate whether male *P. cinereus* can discriminate between females based on chemical cues. The trial pairings used in our visual preference experiment were repeated in this chemical experiment. Males and females were each used in multiple trials, but no male experienced the same sensory cues from the same female more than once. Although this means that each male encountered the same female twice, the cue he experienced (visual or chemical) differed between encounters. These trials took place in a covered Y‐maze equipped with a low powered air pump which simultaneously provided airflow over both female stimuli in the direction of the focal male (Figure 2b). The shorter arms of the Y‐maze measured 5 L × 5 W (in cm) and served as locations for the female odor sources in this experiment (one arm per female scent). The main arm of the maze measured 10 L × 5 W (in cm) and served as the focal male chamber. Air was supplied from a central pump (Top Fin mini air pump, PetSmart Inc.) attached to a silicone airline tube that we then split to attach to each of the two shorter arms of the arena. Each of these arms was equipped with an individual flow meter to monitor air flow. To ensure equal air flow to each arm, both flow meters were stabilized to 0.03 m^3^/hr before initiating a trial. Female chemical secretions were obtained immediately prior to a trial by collecting a small (ca. 1 L × 2 W, in cm) nonsoiled (feces‐free) portion of the filter paper substrate from each female's home enclosure using latex gloves and clean scissors. These two samples (one per female) were then placed in the shorter arms of the Y‐maze (one per arm). At the start of a trial, the focal male was placed into the arena at the far end of the long arm under an opaque barrier to acclimate for 5 min. At the end of this acclimation period, the barrier was removed to allow the focal male to investigate the chemical cues within the two female chambers for a period of 10 min. A single observer (KEJ) observed each male during this period and recorded his TIP and NT behaviors, again using TrueBasic Event‐PC software. As with our visual experiment, we considered the female with the greatest TIP as the preferred female in that trial and maintained the same low‐light conditions for all trials. At the end of each trial, we thoroughly cleaned the Y‐maze with 70% ethanol to remove any chemical cues prior to re‐use.

### 2.5 Fecundity and reproductive phenology

We housed our sample (*n* = 26) of gravid females in separate enclosures (same dimensions as above) for the duration of our experiment. Females were provided food as described above and checked daily for egg development and deposition. For each female, we estimated the duration of time in days until egg deposition (relative to capture date), the date of egg deposition, and the total number of eggs laid. In addition to providing valuable life history data for our population of *P. cinereus* (and likely others in the region), these data will be useful for evaluating the relationship between the relevant female trait (in this case, her SVL) and her fecundity (i.e., egg number; see Pincheira‐Donoso & Hunt, 2017). This assessment provides a critical physiological link often lacking in studies inferring fecundity selection from mate preference experiments or data on female‐biased sexual size dimorphism alone (e.g., Jones, Monaghan, & Nager, 2001; Marvin, 2009).

### 2.6 Statistical analysis

We conducted a total of 288 trials (144 trials per experiment). In both experiments, a trial was discarded if the focal male did not move within the first eight minutes of the trial. We attempted to re‐do discarded trials once; however, if the male did not move in the repeated trial, the trial was discarded completely from analysis. A total of three visual and four chemical experiment trials were discarded, resulting in 141 visual and 140 chemical trials in our final experimental datasets. All statistical analyses were conducted in the R 3.0.2 software environment (R Core Team, 2014), and all means are provided ±1 standard error (*SE*) unless otherwise noted.

We tested for differences in SVL and mass by sex and morph (females only) using separate *t*‐tests. For mass, we used the mass estimated at the start of our behavioral experiments. We note that although mass of all salamanders increased from capture during our acclimation period (mean ± *SE*: 0.08 ± 0.01 g), sex differences in mass were preserved (*t* = 4.2, *df* = 104.816, *p* < 0.001), and for females, mass changes were not morph specific (*t* = 1.2, *df* = 69.882, *p* = 0.24).

Females in each trial were scored as “preferred” or “not preferred” based on male TIP behavior: The female that received more attention from the focal male (=greater TIP) was labeled the preferred female of that trial. This binary preference assignment was robust, as males spent over twice as much TIP to preferred females compared to not‐preferred females (visual experiment, winners: 294.7 ± 8 s, losers: 134.4 ± 6.5 s, paired *t*‐test, *t* = 13.19, *df* = 140, *p* < 0.001; chemical experiment, winners: 276.4 ± 8.3 s, losers: 101.9 ± 6.3 s, paired *t*‐test, *t* = 16.01, *df* = 139, *p* < 0.001). For each experiment, we used separate chi‐squared tests to evaluate any side bias (i.e., whether salamanders consistently preferred the left or right side of the arena) and whether preferences were morph specific, respectively. We evaluated the effect of female traits (SVL, color scores [PC1 and PC2]) and NT on male preference (preferred or not‐preferred) using Bradley‐Terry models for paired comparisons (function “BTm” in package bradleyterry2, Turner & Firth, 2012). Bradley‐Terry models are designed to analyze paired contests (e.g., mate preference trials) with binary outcomes while statistically accounting for the repeated use of individuals in multiple trials, thereby enhancing statistical power when sample sizes are limited (Bradley & Terry, 1952; Stuart‐Fox, Firth, Moussalli, & Whiting, 2006). Because female mass and SVL are highly correlated (*r* = 0.72), we estimated female body condition as the residuals of a linear regression of mass over SVL (both traits were log‐transformed) and included this size‐independent term as a covariate in our models in lieu of raw mass. Higher values for this term reflect relatively heavier (better‐condition) females for their given body size. Our models also allowed for interactions between predictors, which allowed us to capture potential but relevant nuances in male preference (e.g., if male NT frequency is dependent on female‐size). Female ID was included in each model as a random effect (e.g., Stuart‐Fox et al., 2006). A separate Bradley‐Terry model was constructed for each experiment. We selected the most parsimonious model for each experiment using a stepwise procedure and Akaike‐Information Criterion (AIC) scores and used the criterion of *p* < 0.1 suggested in Stuart‐Fox et al. (2006) for retaining a variable in our final model. Significance of the most‐parsimonious model compared to the saturated model for each experiment was assessed using a likelihood‐ratio test.

## 3 RESULTS

### 3.1 Morphology and color

**Figure 3:**
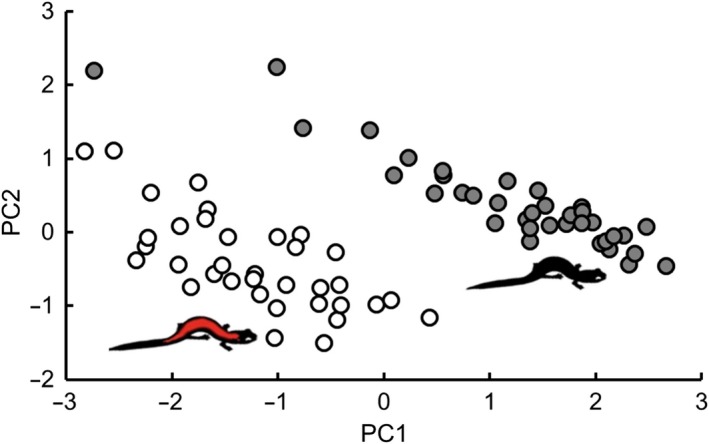
Results of a principal components analysis on the red, green, and blue color channel data extracted from photographs of female striped (*n* = 35, empty points) and unstriped (*n* = 36, filled points) *Plethodon cinereus* used in our male mate preference study. Overall, PC1 and PC2 explained 99.6% of the variation in female dorsal coloration (see Results) [Colour figure can be viewed at http://wileyonlinelibrary.com]

Male *P. cinereus* were smaller in SVL (males: 37.92 ± 0.2 mm; females: 39.86 ± 0.2 mm; *t* = 4.18, *df* = 84.561, *p* < 0.001) and had lower mass (males: 0.73 ± 0.01 g; females: 0.88 ± 0.01 g; *t* = 7.31, *df* = 88.169, *p* < 0.001) than females, reflecting the weak sexual size dimorphism in this species (Blanchard, 1928). At the timing of our experiments, female morphs overlapped in SVL and mass (SVL: *t* = 1.35, *df* = 69.916, *p* = 0.18; mass: *t* = −0.21, *df* = 67.703, *p* = 0.83). In terms of color, PC1 and PC2 explained 80.8% and 18.8% of the variation in female mid‐dorsum coloration, respectively. Overall, PC1 described a dark‐light color gradient (PC1 loadings: red, −0.591; green, −0.634; blue, −0.499) and PC2 described a red‐blue coloration gradient (PC2 loadings: red, −0.515, green, −0.18, blue, 0.838; Figure 3). Striped females had lower loadings on both PC1 and PC2 (PC1: *t* = −10.8, *df* = 62.902, *p* < 0.001; PC2: *t* = 5.4, *df* = 68.818, *p* < 0.001; Figure 3). We detected no correlation between female color score and body size (PC1: *r* = 0.13, *p* = 0.3; PC2: *r* = −0.2, *p* = 0.1) or body condition (PC1: *r* = 0.2, *p* = 0.1; PC2: *r* = 0.04, *p* = 0.8).

### 3.2 Visual experiment

Female pairs in each visual experiment trial differed in both SVL (mean difference = 2.94 mm, range: 0.06–11.13 mm) and mass (mean difference = 0.14 g, range: 0.001–0.551 g). We detected no side bias in our visual experiment (69 left, 72 right; χ^2^ = 0.1, *df* = 1, *p* = 0.8). Of our 141 trials in our visual experiment, striped females and unstriped females were preferred during 76 and 65 trials, respectively. Male *P. cinereus* thus did not prefer a female color morph in this experiment (χ^2^ = 0.86, *df* = 1, *p* = 0.35). Our most parsimonious Bradley‐Terry model for the visual experiment had a significantly better fit to the data than a saturated model (saturated model AIC = 174.04, reduced model AIC = 163.42; χ^2^ = 24.17, *df* = 9, *p* = 0.004). This reduced model only included a single term for NT: specifically, males exhibited more NTs toward preferred females (β_NT_ = 0.448 ± 0.099, *Z* = 4.51, *p* < 0.001). However, no female traits were associated with male preference in this experiment (all *p* > 0.2 in saturated model).

### 3.3 Chemical experiment

Female pairs in each trial differed in both SVL (mean difference = 2.9 mm, range: 0.06–11.1 mm) and mass (mean difference = 0.14 g, range: 0.001–0.551 g). We did not detect any side bias within our chemical preference experiment (61 left, 79 right; χ^2^ = 2.3, *df* = 1, *p* = 0.13). Of the 140 chemical‐preference trials observed, there were 69 striped winners and 71 unstriped winners, and thus, males exhibited no preference for females based on color morph (χ^2^ = 0.03, *df* = 1, *p* = 0.87). Our most parsimonious model had a significantly better fit to our data than a saturated model (saturated model AIC = 131.74, reduced model AIC = 127.49; χ^2^ = 31.56, *df* = 5, *p* < 0.001), and included female SVL, NT, and the interaction between female SVL and NT (the latter of which was nonsignificant, see Table 1). Specifically, striped males were more likely to prefer larger females and exhibited more NT behaviors toward preferred females overall (Table 1).

**Table 1:** Bradley‐Terry model output illustrating effects of female snout‐vent length (mm), number of male nose taps, and their interaction, on male *Plethodon cinereus* mate preference behavior in our chemical experiment. These variables are the best predictors of male preference in our sample (*n* = 140 trials). We assigned preference status (preferred or not preferred) to a female at the end of each trial based on the time the focal male spent in proximity to her scent: the focal male spent more time near the preferred female (see Methods)

Variable	Coefficient	*SE*	*Z*	*p*‐Value
Snout‐vent length	0.219	0.103	2.05	0.041
Nose taps	1.277	0.54	2.37	0.018
Snout‐vent length × Nose taps	−0.025	0.013	−1.95	0.051

### 3.4 Fecundity and reproductive phenology

**Figure 4:**
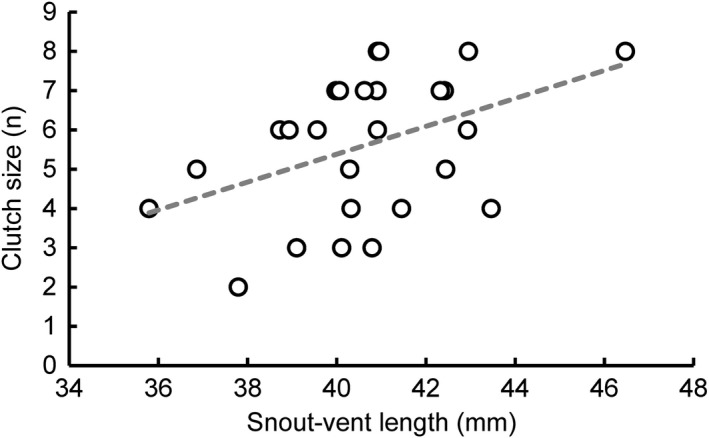
Body size (snout‐vent length, in mm) predicts fecundity (number of eggs) in our population of *Plethodon cinereus* (*n* = 26 females; *p *= 0.027, see Results)

Gravid females averaged 40.66 ± 0.43 mm SVL and laid a median of *n* = 6 eggs (range: 2–8 eggs). Eggs were deposited over a period of 34 days from 10 June–14 July 2013, and began hatching on 5 August, with the last eggs hatched by 25 September 2013. On average, the eggs of a given clutch began to hatch after a period of 66.42 ± 2.56 days. Female body size predicted egg number in our sample (*r *= 0.435, *F*_1,24_ = 5.59, *p *= 0.027). Specifically, larger females produced more eggs (Figure 4). Striped (*n* = 10) and unstriped (*n* = 16) females do not differ in fecundity (*t* = −1.64, *df* = 18.396, *p* = 0.12) or time until hatching (*t* = −0.9, *df* = 13.896, *p* = 0.38).

## 4 DISCUSSION

Although nonrandom mating interactions may contribute to the maintenance and evolution of discrete color polymorphisms within a population, our understanding of the contribution of male mate preferences to this process remains limited. In our mate choice experiments, male *P. cinereus* exhibited more nose tap behaviors toward preferred females, suggesting that this behavior may also be useful for indicating male preferences in this species. However, males only used chemical cues to nonrandomly assess potential mates and exhibited a preference toward larger females in that experiment. We also described a strong positive association between female body size and fecundity in this population; thus, male choosiness has the distinct potential to influence female productivity. Overall, ours is the first study to demonstrate male preference in a terrestrial salamander and to provide evidence that male mate preference may serve as an agent of fecundity selection.

Social interactions in plethodontids are behaviorally complex (Houck & Arnold, 2003; Jaeger, 1984; Jaeger et al., 2016). Previous models of *P. cinereus* mating behavior suggested that this species should match the traditional sexual selection paradigm (e.g., Mathis, 1991), and studies in other parts of their geographic range supported this conclusion (Meche & Jaeger, 2002). However, our study clearly reveals that exceptions to this paradigm occur and may have a strong influence on female reproductive phenology (see Results). In addition, we expected that a genuine male preference should be detectable across all behaviors exhibited by a male. In both experiments, male nose tap frequency was highest toward preferred females (estimated by his time in proximity to a female; see Methods). Nose tap behaviors are exhibited during the early stages of both mating and agonistic interactions to gain information about a potential mate or opponent (Arnold, 1976; Chouinard, 2012; Page & Jaeger, 2004). Future studies of male–female mating interactions in *P. cinereus* could therefore rely on nose tap frequency as a proxy for preference (e.g., Schubert, Houck, Feldhoff, Feldhoff, & Woodley, 2006). Our findings also open the possibility of *in situ* field studies of mating behavior in *P. cinereus*, given the greater ease of measurement of nose taps (i.e., counts) compared to time in proximity.

In general, males only exhibited nonrandom preferences toward females in our chemical experiment. Plethodontid salamanders may rely more heavily on chemical than visual cues for intraspecific communication given their well‐developed olfactory and vomeronasal systems (Houck et al., 2008; Jaeger, 1986; Marco et al., 1998; Mathis et al., 1995). For example, female *P. cinereus* can detect differences in the diet quality of male conspecifics based on chemical secretions alone (Chouinard, 2012), which may confound preferences for one resource (food) for another (mates). In our current study, we avoided any confounding effects of dietary variation on male mating behavior by providing salamanders a constant diet prior to and during experimentation. In terms of reproduction, males have been shown to discriminate between gravid and nongravid females based on chemical cues (Marco et al., 1998), and we show that males can also use these cues to differentiate among nongravid females varying in body size. The composition of female chemical secretions appears to be condition dependent given their covariation with female size (current study) and reproductive state (Marco et al., 1998). A similar link between chemical cues (in feces) and size in male *P. cinereus* has been established (e.g., Mathis, 1990), suggesting that this condition dependence is not sex limited. Energy allocation trade‐offs between chemical production and energetic demands of growth and reproduction may underlie these observed patterns, but more work is needed to assess the validity of this claim.

An additional key goal of our study was to determine whether males can differentiate among the female morphs, which was not the case (see Results). In a previous study on female mate preferences (Acord et al., 2013), female *P. cinereus* exhibited a similar inability to differentiate among the male morphs based on their secretions. Thus, the color morphs may not differ in the composition of their chemical secretions in a controlled setting (females, this study; males, Acord et al., 2013). However, the results of some field studies have been used to infer that the color morphs exhibit assortative mating (e.g., Acord et al., 2013; Anthony et al., 2008). To reconcile these considerations, it may be helpful to draw connections between what is known regarding salamander communication and morph differences in diet and behavior in *P. cinereus*. Chemical cues in salamanders have been shown to signal variation in diet quality (Chouinard, 2012), reproductive state (Marco et al., 1998), and sex (Mathis, 1990), among other qualities. In terms of the *P. cinereus* polymorphism, striped morphs preferentially consume higher quality prey than unstriped morphs (Anthony et al., 2008) and, for males at least, striped individuals are also more aggressive (Reiter et al., 2014) and defend higher‐quality territories (Anthony et al., 2017). As a result, observations of color assortment in nature may be driven by an interaction among a baseline preference for larger‐sized females by striped males, differences in agonistic, territorial behavior between the male morphs affording striped males access to higher‐quality prey, and a preference by females to attain access to those same higher‐quality resources (assessed via male chemical or fecal cues; Walls et al., 1989). Alternatively, differences in aggression alone may contribute to assortment, as striped morphs of both sexes would co‐defend similar high‐quality resources (Lang & Jaeger, 2000), and thus be more likely to interact (Acord et al., 2013). Regardless, for field studies, morph diet differences can confound interpretations of mate preference (see Karuzas, Maerz, & Madison, 2004) in addition to the inherent difficulty of preventing chemical communication between focal salamanders in a natural setting. Consequently, more work is needed to evaluate whether females rely on visual cues to select mates or if color assortment is a byproduct of the interaction suggested above.

Male preference for larger females should be adaptive in *P. cinereus* because laboratory cross‐fostering experiments reveal that larger mothers (foster or genetic) produce larger‐sized hatchlings, likely mediated via extended parental contact with developing eggs (Crespi & Lessig, 2004). In our current study, this male preference, coupled with the positive covariation between female size and fecundity, provides evidence that male preference is an agent of fecundity selection in *P. cinereus* (Pincheira‐Donoso & Hunt, 2017). To date, previous studies have inferred the possibility of fecundity selection in salamanders, based on data drawn from different species that suggests that (a) female size covaries with fecundity (Lotter, 1978; Nagel, 1977) and (b) males may prefer to pair with larger, presumably more fecund females in the wild (for *P. cinereus* see Acord et al., 2013; Anthony et al., 2008; for other plethodontids see Verrell, 1985, 1989). To our knowledge, ours is thus the first study to demonstrate the critical links between male preference and female size, and female size and fecundity, in a single population of *P. cinereus*. Our study also reveals that the female morphs do not differ in fecundity or body size, although in general, females are larger than males (see Results). Male mating behavior may therefore also contribute to the weak female‐biased sexual‐size dimorphism reported for this species (Acord et al., 2013; Nagel, 1977; Werner, 1971; this study).

## 5 CONCLUSIONS

Interest and appreciation of the role of male mate preferences in mating interactions has grown over the past several years, revealing a growing consensus that males also contribute to the maintenance and evolution of phenotypic diversity in animal populations. For color polymorphic species, morph‐specific mate preferences can also enhance rates of ecological divergence among syntopic color morphs (Gray & McKinnon, 2007). Although male mate preferences in our study were not female morph specific, they should nonetheless contribute to observed *P. cinereus* spatial dynamics in the wild (see Anthony et al., 2008). In a broader sense, the combined lack of male or female (Acord et al., 2013) morph‐specific mating preferences in our study system reveal that factors other than mate choice may drive color polymorphism evolution. Interestingly, our findings also provide evidence for fecundity selection in a clade where this process has been inferred to occur for at least four decades (e.g., Salthe, 1969). Other studies have suggested that females may be under selection by male mating behavior (e.g., Clutton‐Brock, 2007; MacLeod & Andrade, 2014; Svensson, Lehtonen, & Wong, 2010) and given that we detected a clear preference for female body size in an aggressive and territorial (and thus high quality) male morph, this may also be the case for *P. cinereus* (Fitzpatrick & Servedio, 2017). However, our reproductive phenology data support that female body size is most likely under fecundity, and not sexual (emphasized in the above studies) selection, and that male preferences are an important contributor to this process.

## CONFLICT OF INTEREST

None declared.

## AUTHOR CONTRIBUTIONS

KEJ, CDA, and CMH designed and conducted the experiment, and MSL analyzed and interpreted the data. MSL and KEJ wrote the initial draft of this manuscript, and all authors contributed to revisions. All authors and approved the final version.

## DATA ACCESSIBILITY

Data from this study will be archived in the Dryad Digital Repository upon publication.
